# Daily Torpor and Sleep in a Non-human Primate, the Gray Mouse Lemur (*Microcebus murinus*)

**DOI:** 10.3389/fnana.2019.00087

**Published:** 2019-09-24

**Authors:** Julie Royo, Fabienne Aujard, Fabien Pifferi

**Affiliations:** UMR CNRS MNHN 7179 MECADEV, BioAdapt Team, Brunoy, France

**Keywords:** gray mouse lemurs, EEG, sleep, torpor, body temperature

## Abstract

Daily torpor is an energy-saving process that evolved as an extension of non-rapid eye movement (NREM) sleep mechanisms. In many heterothermic species there is a relation between torpor expression and the repartition of the different behavioral states of sleep. Despite the presence of sleep during this period of hypothermia, torpor induces an accumulation of sleep debt which results in a rebound of sleep in mammals. We aimed to investigate the expression of sleep-wake rhythms and delta waves during daily torpor at various ambient temperatures in a non-human primate model, the gray mouse lemur (*Microcebus murinus*). Cortical activity was measured with telemetric electroencephalography (EEG) recordings in the prefrontal cortex (PFC) during the torpor episode and the next 24 h following hypothermia. Gray mouse lemurs were divided into two groups: the first group was subjected to normal ambient temperatures (25°C) whereas the second group was placed at lower ambient temperatures (10°C). Contrary to normal ambient temperatures, sleep-wake rhythms were maintained during torpor until body temperature (Tb) of the animals reached 21°C. Below this temperature, NREM and REM sleep strongly decreased or were absent whereas the EEG became isoelectric. The different states of sleep were proportional to Tb_min_ during prior torpor in contrast to active phases. Delta waves increased after torpor but low Tb did not induce greater delta power compared to higher temperatures. Our results showed that Tb was a determining factor for the quality and quantity of sleep. Low Tb might be inconsistent with the recovery function of sleep. Heterothermy caused a sleep debt thus there was a rebound of sleep at the beginning of euthermia to compensate for the lack of sleep.

## Introduction

Sleep is a regulated recovery mechanism (Tilley et al., 1987; Tononi and Cirelli, 2006), the timing of which is gated by the circadian clock that modulates its expression according to endogenous and exogenous factors such as nutritional status or light (Edgar et al., 1993; Wyatt et al., 1999). This state can be characterized by different behavioral criteria such as relative inactivity accompanied by a loss of consciousness, reduced responsiveness to external stimulation, decreased homeostasis and a rapid reversibility (Zimmerman et al., 2008). This last point differentiates sleep from coma or anesthesia (Campbell and Tobler, 1984). Extended wakefulness periods induce an increase in sleep need related to the duration of prior wakefulness and this sleep pressure is dissipated during the next sleep period (Tobler, 2005). Sleep plays an important role in different processes such as synaptic plasticity and memory functions (Tononi and Cirelli, 2014) and metabolic functions and energy balance (Schmidt, 2014). The amount and nature of sleep is vary according to age, size and ecological factors (Siegel, 2005). In mammals, sleep is divided into two broad types: non-rapid eye movement (NREM) and rapid eye movement (REM) sleep (Rial et al., 2010; Roebuck et al., 2014). These two types of sleep occur alternatively in cycles throughout the night (Tobler, 1995). During NREM sleep, biological functions, such as metabolism, are reduced, and energy is allocated to repair cellular damages, transform recently encoded neuronal memory representations for integration into long-term memory and reorganize neural networks (Tononi and Cirelli, 2003, 2014; Palchykova et al., 2006; Aton et al., 2009; Rasch and Born, 2013; Schmidt, 2014; Spaeth et al., 2015). In contrast, REM sleep is characterized by important cerebral activity as the brain is in a waking state and appears to perform select brain functions after the recovery process (Vyazovskiy and Delogu, 2014) and stabilize transformed memory (Rasch and Born, 2013). Sleep can reduce the cumulative energy demands and cellular stress produced by wakefulness but it can also allocate daily energy use more efficiently (Schmidt, 2014). Indeed, during sleep, energy resources are preferentially allocated to biological mechanisms (i.e., growth, immune functions or cellular repair), contrary to wake processes, important consumers of energy, which are inhibited (i.e., vigilance, foraging or reproduction). These different findings show that sleep is an energy-saving mechanism.

When faced with harsh environmental conditions, many endothermic species exhibit a seasonal heterothermy, which is usually divided into two types: daily torpor, lasting less than 24 h, and hibernation, lasting more than 24 h (Génin and Perret, 2003; Geiser, 2011, 2013; Ruf and Geiser, 2015). These mechanisms are composed of different states (Kilduff et al., 1993; Heldmaier et al., 2004). First, the entrance into heterothermy causes a rapid reduction in metabolic rate, which precedes a decrease in body temperature (Tb). Then, deep hypometabolism can be maintained for several hours (daily torpor) to several weeks (hibernation). This state is achieved by induced metabolic inhibition during the entrance into heterothermy but also by the slowdown of metabolic reactions through the thermodynamic effect of hypothermia. Finally, the event is terminated by an arousal that depends on the activation of non-shivering or shivering thermogenesis (Cannon and Nedergaard, 2004), and animals rapidly raise their body temperature to euthermic levels (Ortmann and Heldmaier, 2000; Heldmaier et al., 2004). The energy-savings obtained by daily torpor and hibernation are on the order of approximately 60–70 and 90% (Ruf and Geiser, 2015), respectively.

Heterothermy seems to have the appearance of sleep: sleep-like posture and a reduction in locomotor activity and Tb (Heller and Ruby, 2004). However, this process has an impact on the sleep-wake cycle, especially on NREM and REM sleep. Several electroencephalographic (EEG) studies have shown that heterothermy in animals typically starts through NREM sleep (Florant et al., 1978; Walker et al., 1979; Berger, 1984; Deboer and Tobler, 1994). Similar to hibernation, the time spent in REM sleep is reduced or absent at a Tb below 21–25°C (Krilowicz et al., 1988; Strijkstra et al., 1999). EEG studies in hibernators demonstrate that hibernation is characterized by recurring bouts of heterothermy interrupted by euthermic periods of NREM and REM sleep suggest that a sleep debt accumulates during hibernation (Daan et al., 1991; Trachsel et al., 1991; Kilduff et al., 1993; Strijkstra and Daan, 1997; Palchykova et al., 2002). These results suggest that euthermy is necessary for REM sleep and that the occurrence of NREM sleep seems to be correlated with metabolic rate and Tb (Krystal et al., 2013). The arousal episodes may be necessary to induce sleep processes. Moreover, previous studies in Djungarian hamsters show that hypometabolism is followed by a period of sleep (Deboer and Tobler, 1994; Vyazovskiy et al., 2017). Contrary to REM sleep, the time spent in NREM sleep increases with the prior heterothermy duration (Strijkstra and Daan, 1997). It is noteworthy that the effects of hypometabolism are similar to those observed after sleep deprivation (Deboer and Tobler, 1994; Vyazovskiy et al., 2017). Indeed, after the emergence from daily torpor, animals show a rebound of sleep, which is explained by the accumulation of a sleep debt, suggesting that torpor and hibernation might be inconsistent with the recovery function of sleep.

Sleep-wake rhythm was also characterized by the level of delta waves (0.5–4 Hz) during NREM sleep (Borbély and Achermann, 1999; Borbély et al., 2016). This EEG indicator during NREM sleep was proportional to the prior duration of the wake state (Heller and Ruby, 2004). Indeed, it were higher at the beginning of the euthermic period and then, it gradually decreased during the late stages of arousal. In Djungarian hamsters, during the first hour after emergence, animals entered a NREM sleep characterized by an increase in delta waves (Vyazovskiy et al., 2017). It has been proposed that the role in delta waves may reflect recovery processes typically associated with sleep (Vyazovskiy et al., 2017). Different hypotheses try to explain the cause of the increase of delta waves following torpor arousal, but they are debated: the thermoregulatory hypothesis (García-Allegue et al., 1999), the brain energy hypothesis (Galster and Morrison, 1975; Nizielski et al., 1989; Nestler, 1991; Benington and Heller, 1995) and the synaptogenesis hypothesis (Popov and Bocharova, 1992; Strijkstra et al., 2003; Arendt and Bullmann, 2013; Horowitz and Horwitz, 2019). The most convincing hypothesis is that the changes in delta waves after hypothermia would be associated with structural changes at the neuronal network level (Arendt and Bullmann, 2013; Horowitz and Horwitz, 2019). Studies have shown that during NREM sleep, neurons alternate between periods of depolarization and hyperpolarization (Steriade et al., 1993; Vyazovskiy et al., 2009), which are correlated with delta power (Vyazovskiy et al., 2009). Neuronal populations tend to be more frequently inactive during NREM sleep after torpor due to an overall decrease in network activity (Sanchez-Vives and McCormick, 2000; Haider et al., 2006). Hypothermia would cause a loss of neuronal connections, which would be restored by an increase in delta waves during euthermia (Larkin and Heller, 1998; Strijkstra and Daan, 1998). During deep torpor, ground squirrels showed a reduction in hippocampal dendritic connections (Ruediger et al., 2007; von der Ohe et al., 2007), and these connections were restored within 2 h after arousal (Popov and Bocharova, 1992; von der Ohe et al., 2006). Sleep after hypothermia was a model to investigate the activation mechanisms of neuronal networks and could be a possible interpretation of torpor effects on sleep-wake rhythms.

The goal of this study was to investigate the relation between daily torpor and the expression of sleep at various ambient temperatures in a heterotherm non-human primate exhibiting seasonal daily hypometabolism. The study was performed in the gray mouse lemur (*Microcebus murinus*), a nocturnal primate, endemic to Madagascar, weighing between 60 and 120 g, with a lifespan of 8–10 years in captivity (Languille et al., 2012). This species exhibits different adaptive strategies to survive the 6 months of the dry season. During this season, animals spontaneously enter into daily torpor to save energy (Génin and Perret, 2003; Giroud et al., 2008; Canale et al., 2011). We hypothesized that daily torpor disrupts sleep, limiting its recovery function and that the accumulation of a sleep debt causes a rebound of sleep after a torpor bout. To test this hypothesis, we examined the impact of torpor on sleep-wake rhythms in gray mouse lemurs using EEG in the prefrontal cortex (PFC) during episodes of hypothermia compared to normothermia at different ambient temperatures (25°C and 10°C). Then, we investigated the impact of hypothermia on delta waves during torpor bouts and during the subsequent euthermic periods.

## Materials and Methods

### Animals and Housing Conditions

Six male gray mouse lemurs (*M. murinus*) born and raised in the laboratory colony of UMR 7179 (CNRS/MNHN, France, license approval n° A91.114.1) were studied. All experimental procedures were approved by the ethical committee “Comité d’éthique Cuvier” (authorization n°68–018). Animals were maintained in individual cages with branches and wooden nests at constant temperature (24–26°C) and relative humidity (55%). In the housing environment, gray mouse lemurs were handled during the winter-like short day length photoperiod (10:14 h light:darkness). Animals were fed fresh fruits and a mixture of cereals, water, banana milk and eggs prepared daily in the laboratory.

### EEG Recording Set up and Surgery

A wireless telemetry system (Data Science International, DSI, St. Paul, MN, USA) was used to collect physiological data such as locomotor activity, Tb, EEG and EMG signals for extended periods of time while animals were left undisturbed in their cages. The setup consisted of implanted electrodes (silicon elastomer insulated stainless-steel wires, Ø 0.3 mm), a radio transmitter (PhysioTelF20-EET, DSI), and a receiver plate placed below the cage and connected to a personal computer running the data acquisition software Dataquest Lab Pro v.3.0. (DSI). Tb (resolution: 0.05°C), locomotor activity, EEG and EMG signals (500 Hz sampling rate) were simultaneously acquired for each animal.

Surgical implantations were conducted in conditions of sterility, under veterinarian supervision. The surgical procedure was previously described in several references (Pifferi et al., 2012; Royo et al., 2018). The epidural electrode tips were placed over the PFC (2–3 mm from the midline and 7.5 mm anterior to the interneural line, corresponding to the transversal section shown in plate 33 of the “Stereotaxic atlas of the brain gray mouse lemur” Bons et al., 1998), and EMG electrode wires were placed on the neck muscles with a non-absorbable polyamine suture. At the end of surgery, an anti-inflammatory drug was administered subcutaneously (meloxicam, 0.2 mg/kg).

After surgery, the animals returned to their individual cages. The day after surgery, a subcutaneous injection of painkiller and anti-inflammatory drug (meloxicam, 0.2 mg/kg) was administered. Each day, the recovery of Tb, locomotor activity, EEG signal stabilization and body weight were checked. At least 4 days were allowed for recovery before the beginning of recordings. The implant was removed 2–3 weeks after the first surgery.

### Experimental Protocol

Four animals were maintained at constant temperature (24–26°C), whereas two gray mouse lemurs were subjected to a variation of ambient temperature. They were acclimated to the experimental device for 5 days at 25°C and then exposed to a cold environment (17 days at 10°C). The ambient temperature gradually increased until the end of the study. Gray mouse lemurs exhibiting torpor bouts with Tb <33°C (Génin and Perret, 2003; Canale et al., 2011) were selected for EEG analysis. Torpor was characterized by minimum Tb (Tb_min_, in °C) and torpor bout duration (D_torpor_, in min). Then, to compare torpor to non-torpid conditions at 25°C, a day without torpor in the same animal, the day before at the same time period, served as control (pre-torpor condition).To determine the impact of torpor on the sleep-wake cycle and sleep rebound, three conditions were analyzed: pre-torpor, during torpor and post-torpor.

### EEG Data Analysis

EEG, EMG and Tb signals were continuously acquired for 12–13 consecutive days. Behavioral states were scored by visual inspection of the signals in 10-s epochs to determine the periods of activity, with the aid of the Neuroscore software v.2.1. (Data Science International), over a period corresponding to the day with an episode of torpor and the control day in the same animal corresponding to the day without a torpor bout. The following behavioral states were discriminated (Grigg-Damberger, 2012): NREM sleep, REM sleep, quiet wake (W) and active wake (A; Supplementary Figure S1). The waking state was characterized by a low-amplitude, high-frequency EEG pattern and phasic EMG activity (Supplementary Figures S1A,B). During REM sleep, the EEG pattern was similar to waking state but there was a muscular atony (Supplementary Figure S1C). First, an approach to study NREM phases in all animals allowed us to observe the existence of only two phases of NREM sleep in gray mouse lemur NREM 1 and NREM 2 (Supplementary Figures S1D,E). NREM 1 is the lightest stage of sleep, characterized by slowed frequency, an increased amplitude of the EEG pattern compared to the waking state and low EMG activity. NREM 2 represents a deeper sleep characterized by the occurrence of high-amplitude slow-waves, low EMG activity and the presence of K-complexes and sleep spindles that are signs of progression into sleep (De Gennaro and Ferrara, 2003; De Gennaro et al., 2005; Bellesi et al., 2014). At Tb below 21°C, EEG became isoelectric, which and this change characterized by a complete loss of cortical electrical activity in EEG recordings (Supplementary Figure S1F).

Delta power analysis was performed on the EEG data with custom MATLAB scripts (The MathWorks Inc., Natick, MA, USA) based on the Welch method with a 0.1 Hz frequency resolution over the 0.5–4 Hz range. The total duration of each episode for different conditions (control, torpor, pre-torpor and post-torpor) was analyzed. Delta power data were subjected to decimal logarithm transformation.

### Statistical Analysis

All data were analyzed with R (version 3.5.2.) and were expressed as median ± interquartile range. We started by extracting some parameters from the torpor: Tb_min_ and D_torpor_. To determine the relationship between parameters, we used Pearson’s correlation. The two groups of torpor were compared with Student’s *t*-tests with a Welch correction. All data of vigilance state distribution were analyzed with a linear mixed model for repeated measures with “animal” and “torpor” or “ambient temperature” (control/torpor group) factors. First, we scored vigilance state repartition during 24 h by visual inspection. Then, a separation of data was made to study daytime and nighttime. Finally, we performed statistical analysis on vigilance state distribution during the episode of torpor and the post-torpor period at 25°C and 10°C compared to the control and pre-torpor conditions. We analyzed the groups by pairwise comparisons (Control/Pre-torpor conditions vs. Torpor/Post-torpor conditions at 25°C; Control/Pre-torpor conditions vs. Torpor/Post-torpor conditions at 10°C; Torpor/Post-torpor conditions at 10°C vs. Torpor/Post-torpor conditions at 25°C). Then, Spearman’s correlation was performed to determine the effect of Tb_min_ on each behavioral state during each time period. Outliers for each sleep-wake phase were identified with Dixon’s *Q* test and the values for the entire event were rejected.

## Results

### Torpor Features

**Figure 1:**
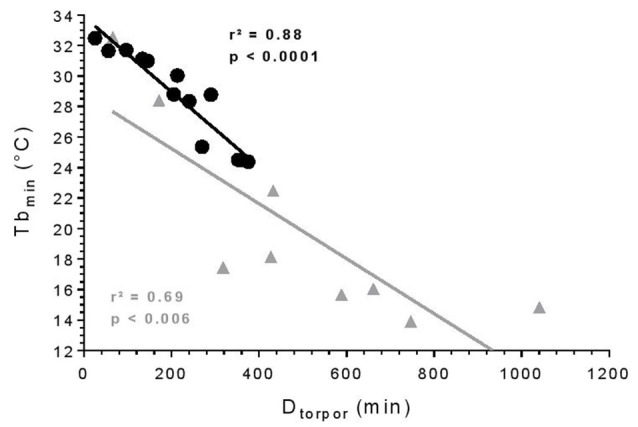
Torpor features. Relationship between minimum body temperature (Tb_min_) and duration of torpor bouts (D_torpor_). Each symbol represents an episode of torpor (25°C: black circle; 10°C: gray triangle). The straight line depicts the linear regression line. *R*- and *p*-values: Pearson’s correlation (25°C: *n* = 4, number of events = 13; 10°C: *n* = 2, number of events = 9).

All torpor episodes were analyzed to investigate the relationship between Tb_min_ during torpor and D_torpor_ (Figure 1). During torpor at 25°C, the median Tb_min_ was 28.8°C ± 5.8 and ranged from 24.4 to 32.5°C. Torpor bouts lasted between 24.7 and 375 min with a median D_torpor_ of 213.5 min ± 156.2. At 10°C, torpor was deeper with a median Tb_min_ of 17.4°C ± 6.8 (*t* = 3.74, df = 10.40, *p* = 0.004) and longer with a median D_torpor_ of 432.5 min ± 343.2 (*t* = 2.68, df = 9.65, *p* = 0.02). At low ambient temperature, Tb_min_ ranged from 13.9 to 32.5°C, and episodes lasted between 65 and 746 min. There was a significant correlation between Tb_min_ and D_torpor_ (25°C: *r*^2^ = 0.88, *p* < 0.0001; 10°C: *r*^2^ = 0.69, *p* = 0.006): the longer torpor lasted, the deeper it was.

### Effects of Torpor on Behavioral States

**Figure 2:**
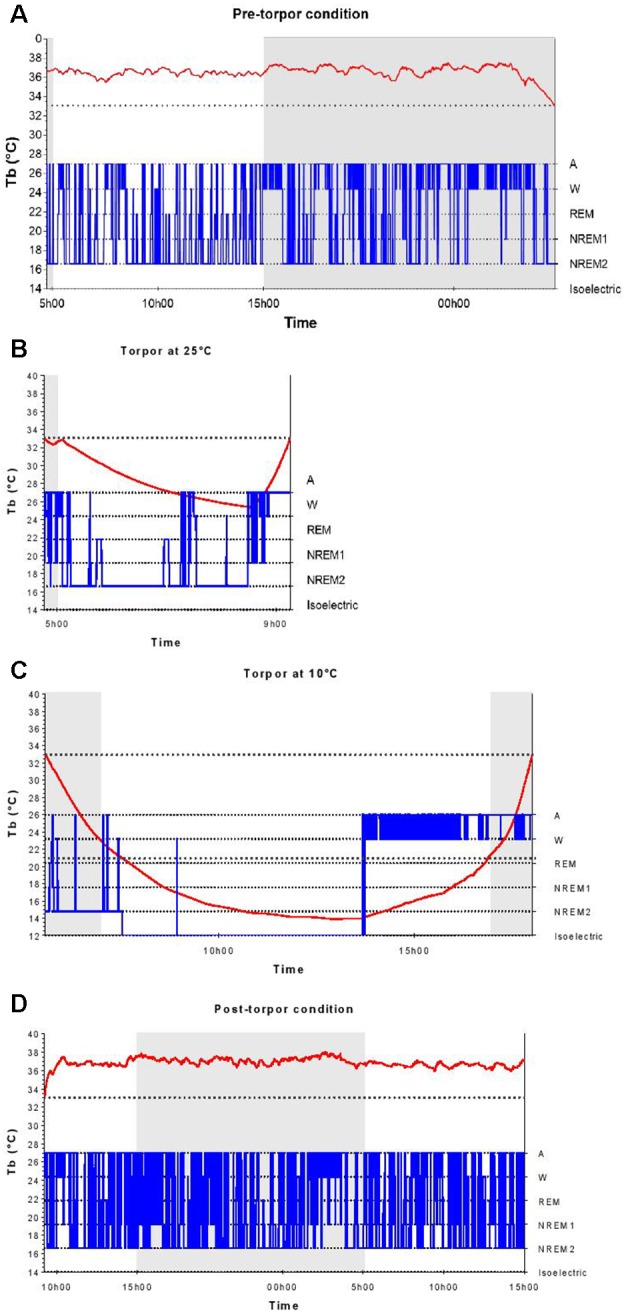
Body temperature and behavioral states during a torpor bout. Time course of body temperature (Tb, red curve) and behavioral states (in blue) during the pre-torpor period **(A)**, the 4.5-h torpor at 25°C **(B)**, the 12.5-h torpor at 10°C **(C)** and the post-torpor period **(D)** in an individual animal. Gray and white rectangles indicate night (dark) and day (light) during periods. The two dotted lines correspond to the threshold value of torpor (33°C) and the Tb value below which the electroencephalography (EEG) is isoelectric (21°C).

In a qualitative analysis of the relationship between torpor and behavioral states, we compared sleep-wake cycles before, during and after a torpor bout. In pre-torpor conditions, we observed an alternation between active and sleep phases without the presence of an isoelectric state (Figure 2A). This fragmentation of activity was also shown in post-torpor conditions (Figure 2D). The majority of torpor bouts started with NREM sleep and ended with wake (Figure 2B). A period of wake generally preceded the rise in Tb. Sleep-wake rhythms were clearly maintained during torpor. At an ambient temperature of 10°C, animals spent most of their time in NREM sleep (Figure 2C). However, when their Tb decreased below 21°C, their EEG became isoelectric.

**Figure 3:**
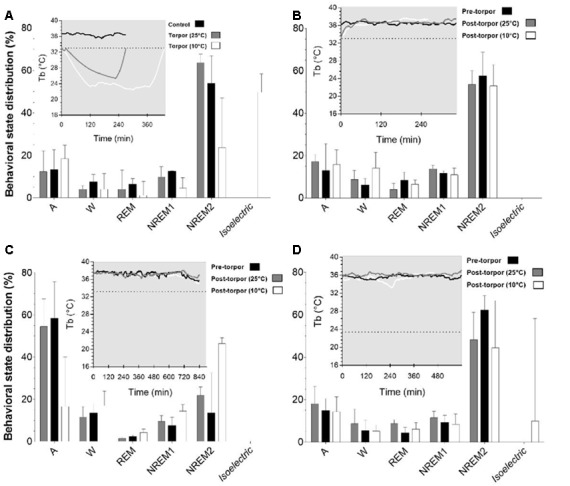
Effect of torpor on behavioral states in gray mouse lemurs. **(A)** Behavioral states percentage in the periods during which torpor was expressed at 25°C (gray) and 10°C (white) and the corresponding control period (black). **(B)** Behavioral state distribution during the light period following a torpor bout (post-torpor at 25°C and at 10°C) and the pre-torpor condition (pre-torpor). **(C)** Behavioral state percentage during the night period following a torpor bout (post-torpor at 25°C and at 10°C) compared to the pre-torpor period. **(D)** Behavioral state distribution during the light period the day after torpor bout (at 25°C and at 10°C) and corresponding pre-torpor period. Each box in the figures shows the body temperature (Tb) in each condition (torpor at 25°C and 10°C, control, pre-torpor and post-torpor at 25°C and 10°C) during the corresponding period. Results are median ± interquartile range. *P* values correspond to mixed-model tests. **(A)** Control and Torpor at 25°C: *n* = 4, number of events = 11, Torpor at 10°C: *n* = 2, number of events = 9. **(B)** Pre-torpor and Post-torpor at 25°C: *n* = 4, number of events = 12, post-torpor at 10°C: *n* = 2, number of events = 8. **(C)** Post-torpor at 25°C: *n* = 4, number of events = 12, post-torpor at 10°C: *n* = 2, number of events = 9, pre-torpor: *n* = 4, number of events = 13. **(D)** Post-torpor at 25°C: *n* = 4, number of events = 11, post-torpor at 10°C: *n* = 2, number of events = 8, pre-torpor: *n* = 4, number of events = 12.

In a quantitative analysis, we analyzed the sleep-wake cycle during torpor (Figure 3A, Tables s 1, 2). At 25°C, no difference in sleep-wake rhythms was observed between torpor and control conditions for the same duration as the episode of torpor. However, when animals were subjected to low ambient temperature (10°C), NREM2 sleep was significantly reduced, while the isoelectric state was increased. Moreover, Spearman’s correlation results suggested that the lower the Tb_min_ was_,_ the lower the NREM and REM sleep and the higher the isoelectric state (Table 3).

**Table 1:** Behavioral state distribution during torpor episodes (at 25°C and 10°C) and the corresponding control periods.

	Control	Torpor at 25°C	Torpor at 10°C
A	13.3 ± 11.8	12.3 ± 12.1	18.5 ± 10.8
W	7.5 ± 5.6	4.1 ± 3.1	4.1 ± 7.8
REM	6.4 ± 5.4	4.0 ± 11.5	1.2 ± 3.9
NREM1	12.5 ± 3.0	9.9 ± 5.9	4.6 ± 5.4
NREM2	53.9 ± 14.3	63.8 ± 13.5	23.6 ± 22.5
Isoelectric	0.0 ± 0.0	0.0 ± 0.0	49.7 ± 55.6

*Values are expressed as median (%) ± interquartile range (Control and Torpor at 25°C: n = 4, number of events = 11; Torpor at 10°C: n = 2, number of events = 9)*.

**Table 2:** Mixedmodel results during torpor episodes (at 25°C and 10°C) and the corresponding control periods.

		Torpor at 10°C		Torpor at 25°C	
		*t*	*p*-value	*t*	*p*-value
Control	A	0.46	0.65	−0.41	0.68
	W	0.52	0.60	−0.01	0.99
	REM	−0.44	0.66	−0.18	0.86
	NREM1	−1.61	0.11	−1.06	0.29
	NREM2	−3.82	<0.001	0.62	0.53
	Isoelectric	2.45	0.01	NA	NA
Torpor at 25°C	A	−0.57	0.57		
	W	−0.12	0.91		
	REM	−0.16	0.87		
	NREM1	0.68	0.49		
	NREM2	2.91	0.04		
	Isoelectric	−2.45	0.01		

*Control and Torpor at 25°C: *n* = 4, number of events = 11; Torpor at 10°C: *n* = 2, number of events = 9*.

**Table 3:** Spearman’s correlation results on the effect of minimum body temperature during torpor (Tb_min_) and behavioral state distribution on torpor episodes (at 25°C and 10°C) and the corresponding control periods.

	*r*_s_	*p*-value
A	−0.20	0.30
W	0.14	0.47
REM	0.33	0.08
NREM1	0.59	<0.001
NREM2	0.42	0.02
Isoelectric	−0.65	<0.001

*Control and Torpor at 25°C: *n* = 4, number of events = 11; Torpor at 10°C: *n* = 2, number of events = 9*.

Then we tried to identify how torpor could affect behavioral states after hypothermia during active and rest periods (Figures 3B–D, Tables s 4–12). During the remaining light period following torpor, we observed a significant increase in quiet wake after hypometabolism compared to pre-torpor conditions (Figure 3B, Tables s 4, 5). REM and NREM1 sleep were significantly decreased in the post-torpor period at 25°C compared to pre-torpor conditions. No difference was observed in active wake state, REM sleep or NREM sleep among post-torpor at 10°C, post-torpor at 25°C and pre-torpor conditions. Tb_min_ during torpor did not have an impact on behavioral states (Table 6). During the night period following hypothermia, torpor at 25°C did not induce differences regardless of sleep-wake state in animals in comparison to the pre-torpor period (Figure 3C, Tables s 7, 8). Lower ambient temperature (10°C) induced significantly decreased activity and increased NREM and REM sleep. When torpor was deeper, the active wake state tended to be reduced and NREM1 sleep tended to predominate (Table 9). Then, the effects of torpor the day after hypothermia were tested to identify potential sleep rebounds (Figure 3D, Tables s 10–12). Torpor did not induce a difference the day after in the active state, while NREM2 sleep was significantly reduced regardless of ambient temperature conditions in comparison to the pre-torpor period (Tables s 10, 11). At 25°C, we observed a significant increase in NREM1 sleep between post-torpor and pre-torpor conditions. After torpor at 10°C, there was an increased isoelectric state when we compared this condition and post-torpor at 25°C or pre-torpor conditions. Indeed, Spearman’s correlation results showed that Tb_min_ induced a decrease in NREM2 sleep and an increase in isoelectric state (Table 12).

**Table 4:** Behavioral state distribution during the light period following a torpor bout (post-torpor at 25°C and 10°C) and the corresponding control period (pre-torpor).

	Pre-torpor	Post-torpor at 25°C	Post-torpor at 10°C
A	12.9 ± 11.6	17.2 ± 4.3	15.8 ± 12.1
W	6.1 ± 6.8	8.8 ± 6.2	14.1 ± 14.1
REM	8.4 ± 7.2	4.1 ± 4.5	6.4 ± 8.2
NREM1	11.6 ± 2.6	13.7 ± 4.5	10.9 ± 6.3
NREM2	57.6 ± 8.6	53.6 ± 7.2	52.9 ± 14.1
Isoelectric	0.0 ± 0.0	0.0 ± 0.0	0.0 ± 0.0

*Values are expressed as median (%) ± interquartile range (pre-torpor and post-torpor at 25°C: *n* = 4, number of events = 12; Post-torpor at 10°C: *n* = 2, number of events = 8)*.

**Table 5:** Mixed-model results during the light period following a torpor bout (Post-torpor at 25°C and 10°C) and the corresponding control period (pre-torpor).

		Post-torpor at 10°C		Post-torpor at 25°C	
		*t*	*p*-value	*t*	*p*-value
Pre-torpor	A	0.27	0.79	0.96	0.34
	W	3.40	<0.001	2.40	0.02
	REM	−1.39	0.17	−3.96	<0.0001
	NREM1	0.15	0.88	2.96	0.003
	NREM2	−0.95	0.34	−1.35	0.18
	Isoelectric	NA	NA	NA	NA
Post-torpor at 25°C	A	0.84	0.40		
	W	−2.27	0.02		
	REM	0.09	0.93		
	NREM1	0.51	0.61		
	NREM2	0.26	0.79		
	Isoelectric	NA	NA		

*Pre-torpor and post-torpor at 25°C: *n* = 4, number of events = 12; post-torpor at 10°C: *n* = 2, number of events = 8*.

**Table 6:** Spearman’s correlation results on the effect of minimum body temperature during torpor (Tb_min_) and behavioral state distribution on the light period following a torpor bout (post-torpor at 25°C and 10°C) and the corresponding control period (pre-torpor).

	*r*_s_	*p*-value
A	−0.09	0.64
W	−0.21	0.27
REM	0.14	0.47
NREM1	−0.09	0.64
NREM2	0.17	0.37
Isoelectric	NA	NA

*Pre-torpor and post-torpor at 25°C: *n* = 4, number of events = 12; post-torpor at 10°C: *n* = 2, number of events = 8*.

**Table 7:** Behavioral state distribution during the night period (14 h) following a torpor bout (post-torpor at 25°C and 10°C) and the corresponding control period (pre-torpor).

	Pre-torpor	Post-torpor at 25°C	Post-torpor at 10°C
A	58.3 ± 33.6	54.6 ± 15.9	16.5 ± 11.7
W	13.4 ± 8.4	11.7 ± 5.4	17.1 ± 12.0
REM	2.4 ± 2.9	1.5 ± 1.9	4.2 ± 1.7
NREM1	7.5 ± 5.9	9.5 ± 5.4	14.4 ± 3.9
NREM2	13.5 ± 20.9	21.8 ± 10.5	46.4 ± 12.9
Isoelectric	0.0 ± 0.0	0.0 ± 0.0	0.0 ± 0.0

*Values are expressed as median (%) ± interquartile range (Post-torpor at 25°C: *n* = 4, number of events = 12; post-torpor at 10°C: *n* = 2, number of events = 9; pre-torpor: *n* = 4, number of events = 13)*.

**Table 8:** Mixed-model results during the night period (14 h) following a torpor bout (post-torpor at 25°C and 10°C) and the corresponding control period (pre-torpor).

		Post-torpor at 10°C		Post-torpor at 25°C	
		*t*	*p*-value	*t*	*p*-value
Pre-torpor	A	−4.25	<0.0001	−0.02	0.98
	W	1.22	0.22	0.09	0.93
	REM	3.83	<0.001	−1.08	0.28
	NREM1	2.61	0.009	0.57	0.57
	NREM2	3.86	<0.0001	−0.25	0.80
	Isoelectric	1.08	0.28	NA	NA
Post-torpor at 25°C	A	4.98	<0.0001		
	W	−1.39	0.16		
	REM	−3.68	<0.001		
	NREM1	−3.75	<0.001		
	NREM2	−4.53	<0.0001		
	Isoelectric	−0.74	0.46		

*Post-torpor at 25°C: *n* = 4, number of events = 12; post-torpor at 10°C: *n* = 2, number of events = 9; pre-torpor: *n* = 4, number of events = 13*.

**Table 9:** Spearman’s correlation results on the effect of minimum body temperature (Tb_min_) during torpor and behavioral state distribution on the night period (14 h) following a torpor bout (post-torpor at 25°C and 10°C) and the corresponding control period (pre-torpor).

	*r*_s_	*p*-value
A	0.34	0.06
W	−0.18	0.33
REM	−0.15	0.42
NREM1	−0.35	0.05
NREM2	−0.27	0.14
Isoelectric	−0.28	0.12

*Post-torpor at 25°C: *n* = 4, number of events = 12; post-torpor at 10°C: *n* = 2, number of events = 9; pre-torpor: *n* = 4, number of events = 13*.

**Table 10:** Behavioral state distribution during the light period (10 h) the day after a torpor bout (post-torpor at 25°C and at 10°C) and the corresponding control period (pre-torpor).

	Pre-torpor	Post-torpor at 25°C	Post-torpor at 10°C
A	14.8 ± 6.7	18.2 ± 14.5	14.3 ± 9.2
W	5.3 ± 6.2	8.7 ± 8.3	5.2 ± 3.2
REM	4.3 ± 2.5	8.8 ± 6.7	6.2 ± 7.8
NREM1	9.4 ± 2.0	11.6 ± 2.9	8.4 ± 10.9
NREM2	62.2 ± 15.3	48.2 ± 13.3	44.4 ± 45.3
Isoelectric	0.0 ± 0.0	0.0 ± 0.0	9.8 ± 55.8

*Values are expressed as median (%) ± interquartile range (post-torpor at 25°C: *n* = 4, number of events = 11; post-torpor at 10°C: *n* = 2, number of events = 8; pre-torpor: *n* = 4, number of events = 12)*.

**Table 11:** Mixed-model results during the light period (10 h) the day after a torpor bout (post-torpor at 25°C and at 10°C) and the corresponding control period (pre-torpor).

		Post-torpor at 10°C		Post-torpor at 25°C	
		*t*	*p*-value	*t*	*p*-value
Pre-torpor	A	−0.38	0.71	1.03	0.30
	W	−0.24	0.81	1.10	0.27
	REM	0.84	0.40	1.43	0.15
	NREM1	−1.29	0.20	2.01	0.04
	NREM2	−2.27	0.02	−2.45	0.01
	Isoelectric	2.91	0.003	NA	NA
Post-torpor at 25°C	A	1.40	0.16		
	W	1.10	0.27		
	REM	0.15	0.88		
	NREM1	1.21	0.22		
	NREM2	0.77	0.44		
	Isoelectric	−2.52	0.01		

*Post-torpor at 25°C: *n* = 4, number of events = 11; post-torpor at 10°C: *n* = 2, number of events = 8; pre-torpor: *n* = 4; number of events = 12*.

**Table 12:** Spearman’s correlation results on the effect of minimum body temperature during torpor (Tb_min_) and behavioral state distribution on the light period (10 h) the day after a torpor bout (post-torpor at 25°C and at 10°C) and the corresponding control period (pre-torpor).

	*r*_s_	*p*-value
A	−0.04	0.84
W	0.19	0.33
REM	−0.07	0.71
NREM1	−0.08	0.70
NREM2	0.42	0.02
Isoelectric	−0.42	0.02

*Post-torpor at 25°C: *n* = 4, number of events = 11; post-torpor at 10°C: *n* = 2, number of events = 8; pre-torpor: *n* = 4; number of events = 12*.

### Effects of Torpor on Delta Waves

All torpor bouts were analyzed to compare the impact of hypothermia on delta waves (Supplementary Figure S2A, Supplementary Table S1A). During torpor, no difference was observed in the frequency bands between 0.5 and 4 Hz regardless of the conditions. Then, we compared the delta power during the pre-torpor and post-torpor conditions to determine the sleep need caused by hypothermia (Supplementary Figures S2B–D, Supplementary Tables S1B–D). During the remaining light period following hypothermia, we observed a significant increase in delta waves during post-torpor at 25°C compared to pre-torpor conditions (Supplementary Figure S1B, Supplementary Table S1B). There were no differences between the post-torpor at 10°C group and the pre-torpor group. Low ambient temperatures (10°C) induced a significant decrease in delta activity in comparison to higher temperatures (25°C). During the night period following a torpor bout, delta waves at 10°C were significantly lower compared to those during pre-torpor (between 0.8 and 4 Hz) and post-torpor at 25°C (Supplementary Figure S2C, Supplementary Table S1C). No difference was observed in delta power between pre-torpor and post-torpor at 25°C. Then, the impact of hypothermia the day after a torpor bout was also tested (Supplementary Figure S1D, Supplementary Table S1D). There were no differences between the post-torpor (10°C and 25°C) and pre-torpor conditions. At 25°C, delta waves were significantly higher between 0.5 and 2 Hz than those of post-torpor at 10°C.

## Discussion

Since several studies suggest that species that perform torpor are deprived of NREM and REM sleep (Harris et al., 1984; Deboer and Tobler, 1994; Krystal et al., 2013), we hypothesized here that daily torpor in the gray mouse lemur disrupts sleep, limiting its recovery function, and that the accumulation of a sleep debt causes a rebound of sleep after a torpor bout. To test this hypothesis, we examined the impact of torpor on sleep-wake rhythms in gray mouse lemurs using EEG in the PFC during episodes of hypothermia and compared it to that of normothermia at different ambient temperatures (25°C and 10°C). As in other heterotherms (Harris et al., 1984; Deboer and Tobler, 1994; Krystal et al., 2013), our results indicate that gray mouse lemurs do sleep during torpor episodes at 25°C. In addition, the presence of sleep during torpor does not necessitate sleep rebound in the hours or day following hypometabolism. However, low Tb disrupts the sleep-wake cycle causing a sleep debt after torpor.

The EEG analysis of behavioral states before, during and after torpor episodes in gray mouse lemur clearly indicates that, at 25°C, this species exhibits both NREM and REM sleep during torpor episodes, with no difference in sleep phases between torpor bouts and control periods. More specifically, animals spend close to 75% of the torpor period in the two stages of NREM sleep (Figure 3A, Tables s 1, 2), which is statistically similar to the value for the corresponding control condition (non-torpid period). Torpor also includes REM sleep stages (~5% of total torpor duration, which is also statistically similar to the value for the control condition). Moreover, Tb has an impact on sleep. When animals are exposed to a cold environment (10°C), NREM and REM sleep decrease during torpor whereas the isoelectric state appears (Table 3).

Moreover, it has been shown in several heterotherms that torpor caused a sleep debt proportional to the prior torpor duration (Strijkstra and Daan, 1997). Here, we demonstrated that in the gray mouse lemur, no sleep rebound was observed after the arousal from torpor bouts and that animals were even more active. Indeed, during the day following torpor, quiet wake increased (+44.3%), whereas REM sleep decreased (−51.2%; Figure 3B, Tables s 4, 5). No differences were observed in the proportion of active and sleep phases between pre-torpor and post-torpor conditions during the first night after torpor (Figure 3C, Tables s 7, 8). However, at low ambient temperature, gray mouse lemurs are less active than during pre-torpor (−71.7%) and post-torpor at 25°C (−69.8%), whereas NREM2 sleep increased (pre-torpor: +70.9%; post-torpor 25°C: +53.0%). NREM1 sleep tended to be correlated with Tb_min_, and prior torpor duration and active wake state were inversely proportional to torpor factors (Table 9). Finally, at 25°C, the next day period showed that gray mouse lemurs spent less time in NREM2 sleep (−29.1%), confirming the absence of sleep debt due to torpor (Figure 3D, Tables s 10, 11). However, gray mouse lemurs subjected to low ambient temperatures exhibited very long-lasting torpors explaining the presence of isoelectric EEG and the decrease in NREM2 sleep even during the following day (the animals were still in torpid state; Table 12).

In hibernating species, the sleep debt is balanced by specific mechanisms (Daan et al., 1991; Trachsel et al., 1991; Strijkstra and Daan, 1997; Krystal et al., 2013; Schmidt, 2014; Blanco et al., 2016; Vyazovskiy et al., 2017). Hibernating animals perform periodic spontaneous elevations of their Tb, which coincide with the euthermic periods of NREM and REM sleep (Daan et al., 1991; Trachsel et al., 1991). This warming, necessary for animal sleep, allows us to see that Tb has an important role in the recovery function of sleep (Deboer and Tobler, 1994). For other species, this sleep debt was reflected by a sleep rebound characterized by an increase in the duration and intensity of NREM sleep and a decrease in REM sleep after arousal from torpor bouts (Strijkstra and Daan, 1997; Schmidt, 2014; Vyazovskiy et al., 2017). Several studies demonstrated that hibernation in *Cheirolageus* showed characteristics in common with non-primate hibernators (Daan et al., 1991; Trachsel et al., 1991; Strijkstra and Daan, 1997; Blanco et al., 2016). These hibernating primates did not sleep during heterothermy but REM and NREM sleep occurred during euthermic periods (Krystal et al., 2013; Blanco et al., 2016). These different results showed that torpor was incompatible with the recovery function of sleep, exhibiting effects similar to those observed after sleep deprivation (Deboer and Tobler, 1994). Animals had to emerge from torpor to be able to fulfil this sleep function. Mouse lemurs did not need to fill a sleep debt because during torpor, sleep achieved its recovery function at 25°C. We can assume that the animals have a deeper and perhaps more effective sleep compared to that in the control condition. Gray mouse lemurs maintain normal circadian rhythms during torpor, which could explain the lack of differences between periods with and without torpor. However, when faced with harsh temperatures, torpor induced sleep rebound after the arousal. Gray mouse lemurs had characteristics similar to other species using this energy-saving strategy. Our results showed that Tb was a determining factor for the quality and quantity of sleep.

Sleep during torpor or hibernation seems to be linked to the Tb reached during hypothermia bouts. Studies in golden-mantled ground squirrels (*Callospermophilus lateralis*) indicate that when hibernation is performed at relatively warm body temperature (22°C), animals do sleep (Walker et al., 1981). When Tb decreases below 21°C, brain activity becomes incompatible with the expression of sleep (Krilowicz et al., 1988; Daan et al., 1991). Such observations suggest that the presence of sleep during hypometabolism is driven by core body (and more specifically brain) temperature. Studies in *C. medius*, a hibernating primate belonging to the same family of cheirogaleids as mouse lemurs, have demonstrated that the presence of sleep during hibernation in this species also depends on Tb. During hibernation at low Tb (down to 10–15°C in this case), NREM sleep is absent in *C. medius* (Krystal et al., 2013). Since body temperature seems to drive the expression of NREM sleep during torpor, it might explain why gray mouse lemurs do sleep during torpor when their Tb are above 21°C whereas they do not sleep below 21°C. When faced with extreme conditions (10°C), animals adapted themselves by drastically reducing their Tb. To conclude, we determined that Tb below 21°C was inconsistent with the recovery function of sleep in *M. murinus*. Animals were in a reversible isoelectric state that was Tb dependent, and this state explained the presence of sleep rebound after torpor.

Here, we investigated for the first time the changes in delta waves caused by hypothermia in gray mouse lemurs. We found that delta waves were not modified by torpor (Supplementary Figure S2A, Supplementary Table S1A). Moreover, torpor induced disturbances after arousal (Supplementary Figures S2B–D, Supplementary Tables S1B–D). Indeed, during the day following torpor, at low ambient temperatures, gray mouse lemurs presented a lower delta activity compared to that in post-torpor at 25°C and pre-torpor conditions (Supplementary Figure S2B, Supplementary Table S1B). No differences were observed in delta power between pre-torpor and post-torpor conditions during the first night after torpor (Supplementary Figure S2C, Supplementary Table S1C). However, at low ambient temperature, gray mouse lemurs showed a decrease in delta waves compared to those at pre-torpor and post-torpor at 25°C. Finally, delta waves were lower at low temperatures in comparison to post-torpor at 25°C (Supplementary Figure S2D, Supplementary Table S1D). One possible interpretation of these results is that torpor tends to change delta waves, reflecting changes in neuronal activity (Vyazovskiy et al., 2009). Several studies showed that during hibernation, different brain areas have reduced neuronal connectivity (Popov and Bocharova, 1992; Hut et al., 2002). Indeed, in hibernating species, synaptic changes were associated with cyclic changes in the density of synaptic vesicles and proteins mediating the rapid rebuilding of dendritic spines and synapses during arousal (von der Ohe et al., 2007; Arendt and Bullmann, 2013). After torpor, a higher delta power was observed, explained by an increase in synaptic strength (Vyazovskiy and Harris, 2013; Tononi and Cirelli, 2014). In this study, we observed that delta waves increased after torpor, but our results showed that low Tb did not induce greater delta power compared to higher temperatures. It is possible that other factors will contribute to specific aspects of network activity. Torpor may be associated with changes in the architecture and activity of cortical networks (Vyazovskiy et al., 2017). During hypothermia, many morphological changes occurred in the brain, inducing a disruption of network connectivity (von der Ohe et al., 2007). Sleep after torpor could help to set up recovery processes. Indeed, during a selective delta-wave deprivation study, Djungarian hamsters showed an increase in slow-waves, reflecting a compensatory homeostatic response.

Conversely to our hypothesis, in gray mouse lemurs, both REM and NREM sleep occur during torpor, at a level equivalent to that of non-torpor periods. However, sleep is temperature-dependent, and low Tb induces an inability to perform REM and NREM sleep. Animals must compensate for the lack of sleep, and thus a sleep rebound occurs. Moreover, Tb also induces an alteration of delta waves after torpor. Other factors must be involved in recovery processes during sleep rebound.

## Data Availability Statement

The datasets generated for this study are available on request to the corresponding author.

## Ethics Statement

This study was carried out in accordance with the recommendations of the ethical committee “Comité d’éthique Cuvier” (authorization n° 68-018). The protocol was approved by the “Comité d’éthique Cuvier.”

## Author Contributions

JR, FA and FP contributed to the conception and design of the study. JR organized the database and performed the statistical analysis. JR wrote the first draft of the manuscript. JR and FP wrote sections of the manuscript. All authors contributed to manuscript revision, read and approved the submitted version.

## Conflict of Interest

The authors declare that the research was conducted in the absence of any commercial or financial relationships that could be construed as a potential conflict of interest.
