# Supplementary material for: Daily Torpor and Sleep in a Non-human Primate, the Gray Mouse Lemur (Microcebus murinus)
Source: Front Neuroanat. 2019 Sep 24;13:87. doi: 10.3389/fnana.2019.00087 (PMC6768945; doi:10.3389/fnana.2019.00087)
Supplement: Supplementary file 1 [file Data_Sheet_1.docx]

**Figure S1: Representative muscular and neuronal signals of different phases of sleep-wake cycle.**

10-s epoch of EEG and EMG recordings during active phase (A), quiet wake (B), REM sleep (C), NREM1 sleep (D), NREM2 sleep (E) and isoelectric (F) periods.


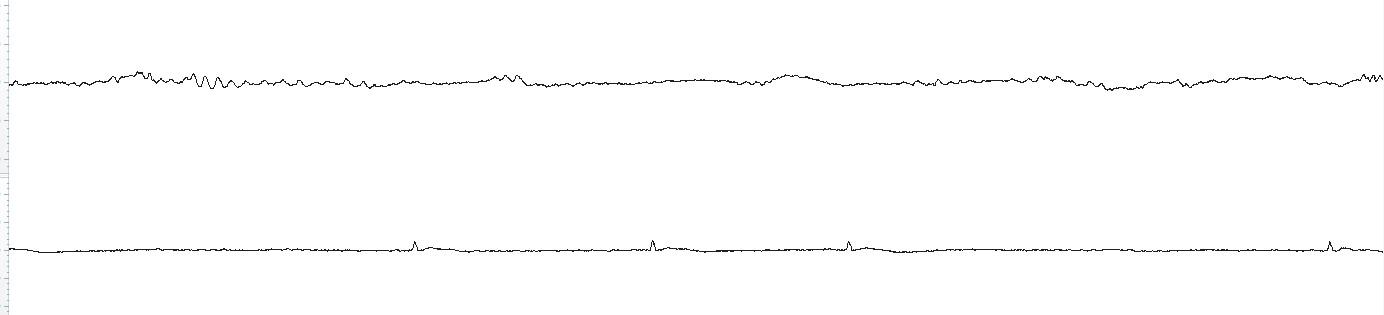

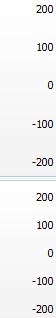

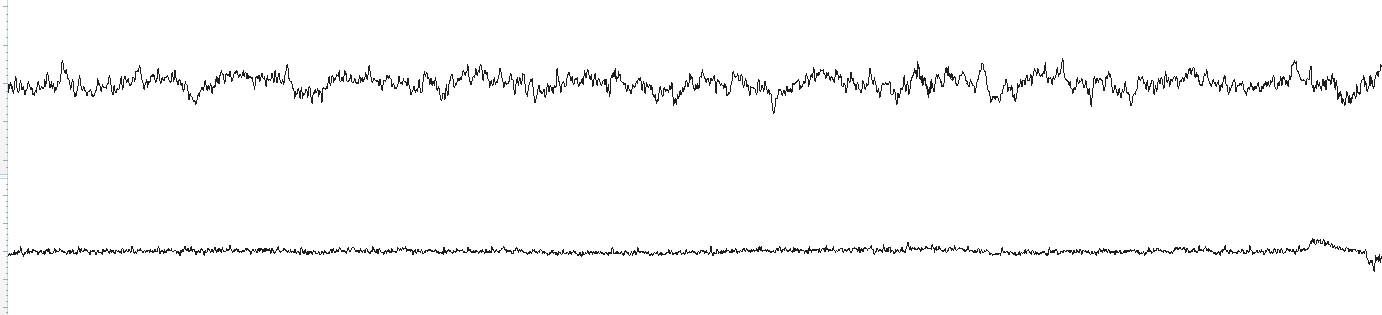

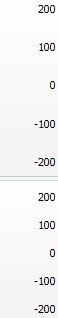

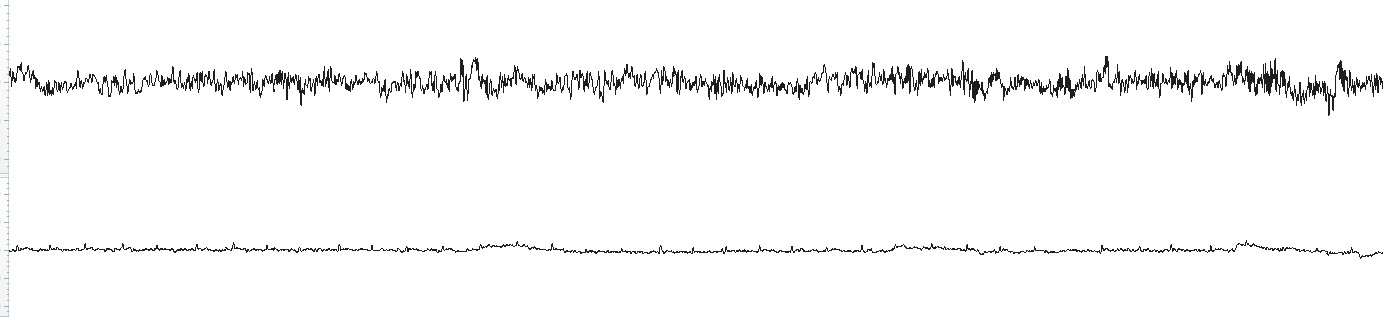

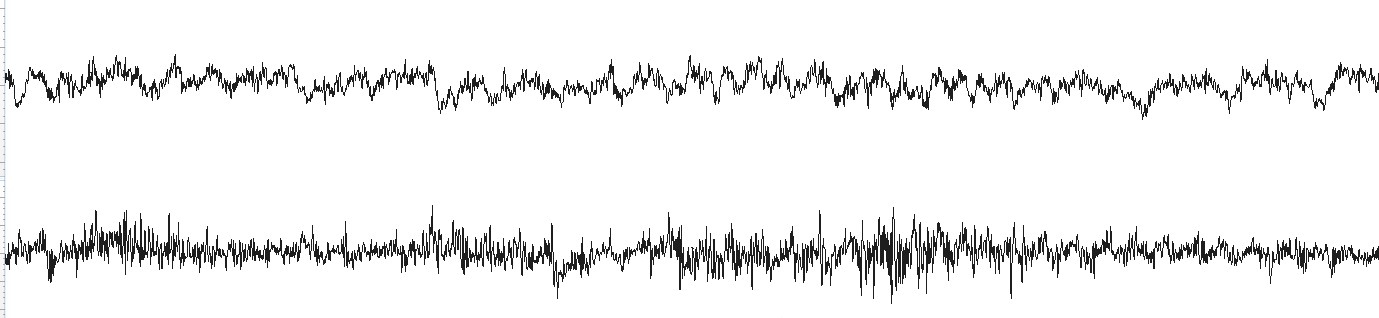

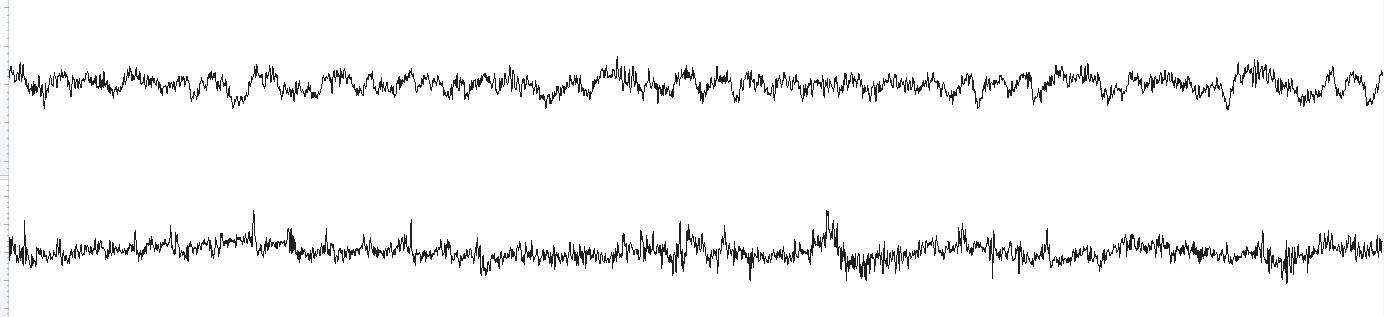

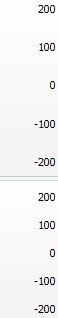

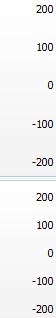

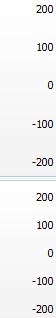

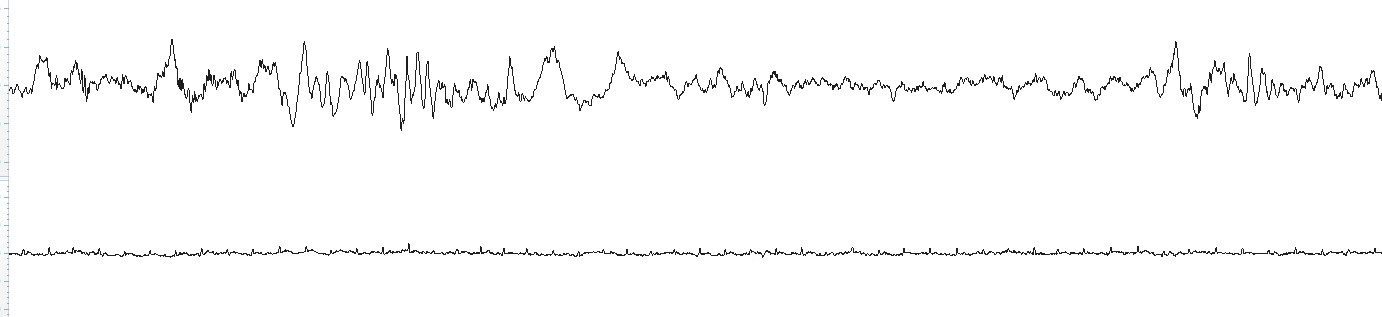

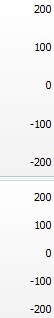

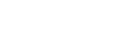


EEG (µV)


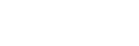

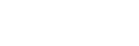


EEG (µV)


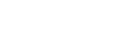

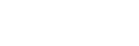


EEG (µV)


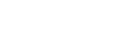

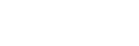


EEG (µV)


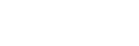


EEG (µV)


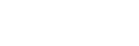

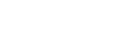


EEG (µV)


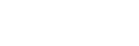


EMG (µV)

EMG (µV)

EMG (µV)

EMG (µV)

EMG (µV)

EMG (µV)

**F**

**E**

**D**

**C**

**B**

**A**

EMG (µV)

EMG (µV)

**Figure S2: Effect of torpor on delta power in grey mouse lemurs.**

A: Delta power logarithms in the periods during which torpor was expressed at 25°C (grey circle) and 10°C (white circle) and the corresponding control period (black circle). B: Delta power logarithms during the light period following a torpor bout (post-torpor at 25°C and at 10°C) and the pre-torpor condition (pre-torpor). C: Delta power logarithms during the night period following a torpor bout (post-torpor at 25°C and at 10°C) compared to the pre-torpor period. D: Delta power logarithms during the light period the day after a torpor bout (at 25°C and at 10°C) and the corresponding pre-torpor period. Results are median. A: Control and Torpor at 25°C: n = 4, number of events = 11, Torpor at 10°C: n = 2, number of events = 9; B: Pre-torpor and Post-torpor at 25°C: n = 4, number of events = 13, Post-torpor at 10°C: n = 2, number of events = 8; C:, Post-torpor at 25°C : n = 4, number of events = 12, Post-torpor at 10°C: n = 2, number of events = 9, Pre-torpor: n = 4, number of events = 13; D: Post-torpor at 25°C: n = 4, number of events = 12, Post-torpor at 10°C: n = 2, number of events = 8, Pre-torpor: n = 4, number of events = 13.


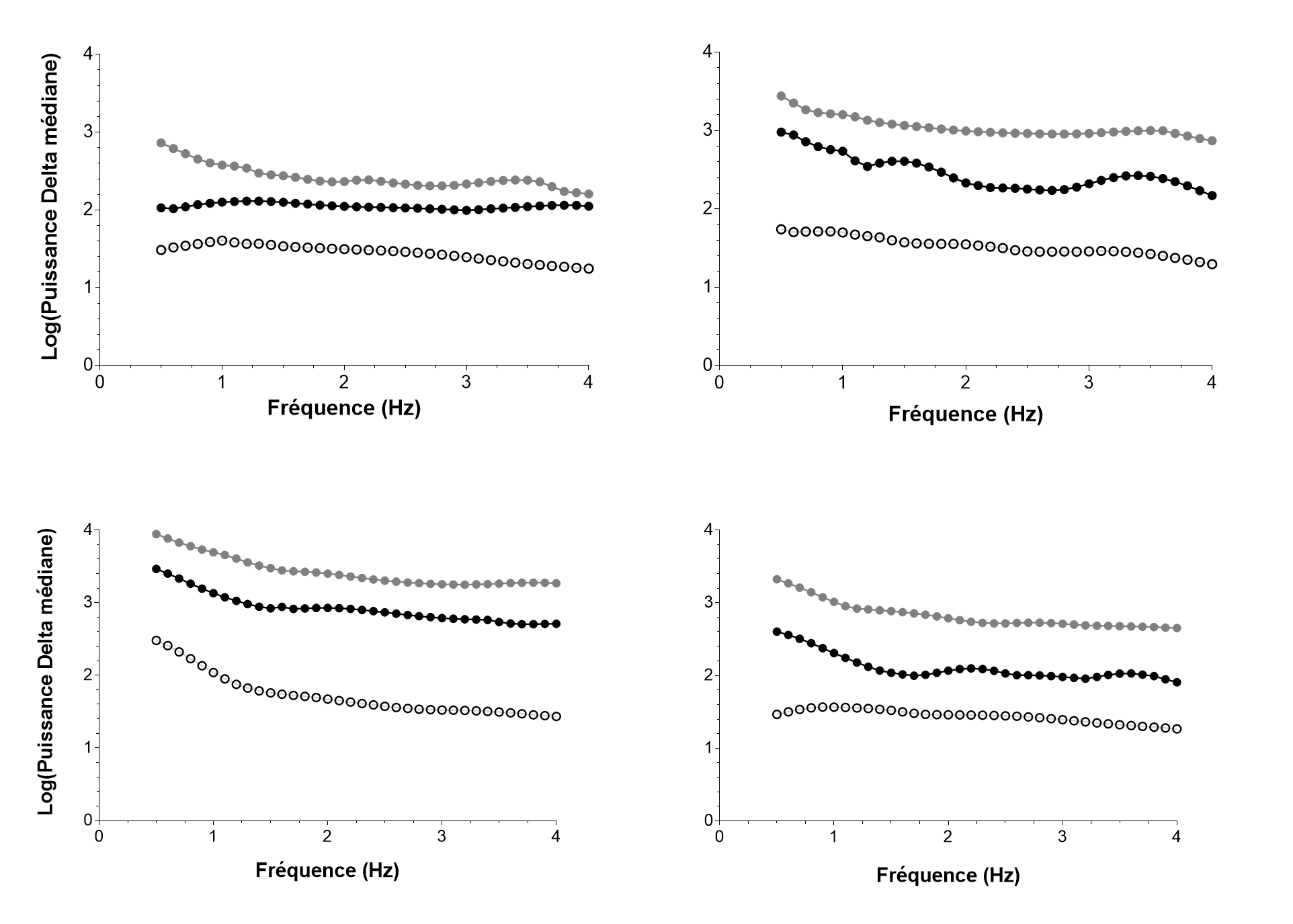


B

A

D

C

**Table S1A: Mixed-model results for delta power during torpor episodes (at 25°C and 10°C) and the corresponding control periods.**

Values are expressed as median (logarithm) ± interquartile range. Control and Torpor at 25°C: n = 4, number of events = 11; Torpor at 10°C: n = 2, number of events = 9.

|  | Control | | Torpor  at 25°C | | Torpor  at 10°C | | Control vs Torpor at 25°C | | Control vs Torpor at 10°C | | Torpor at 25°C vs Torpor at 10°C | |
| --- | --- | --- | --- | --- | --- | --- | --- | --- | --- | --- | --- | --- |
| Frequency  (Hz) | ***median*** | ***IQR*** | ***median*** | ***IQR*** | ***median*** | ***IQR*** | ***t*** | ***p-value*** | ***t*** | ***p-value*** | ***t*** | ***p-value*** |
| 0.5 | 2,03 | 1,84 | 2,86 | 2,38 | 1,49 | 0,29 | - 0.33 | 0.74 | - 1.05 | 0.29 | 1.28 | 0.20 |
| 0.6 | 2,02 | 1,79 | 2,79 | 2,34 | 1,52 | 0,31 | - 0.32 | 0.75 | - 1.04 | 0.30 | 1.25 | 0.21 |
| 0.7 | 2,04 | 1,73 | 2,72 | 2,30 | 1,54 | 0,33 | - 0.30 | 0.76 | - 1.02 | 0.31 | 1.23 | 0.22 |
| 0.8 | 2,07 | 1,69 | 2,65 | 2,27 | 1,56 | 0,30 | - 0.28 | 0.78 | - 0.99 | 0.32 | 1.21 | 0.23 |
| 0.9 | 2,09 | 1,65 | 2,61 | 2,25 | 1,59 | 0,27 | - 0.26 | 0.80 | - 0.95 | 0.34 | 1.20 | 0.23 |
| 1.0 | 2,10 | 1,62 | 2,58 | 2,24 | 1,61 | 0,25 | - 0.24 | 0.81 | - 0.91 | 0.36 | 1.19 | 0.24 |
| 1.1 | 2,11 | 1,59 | 2,56 | 2,25 | 1,58 | 0,28 | - 0.23 | 0.82 | - 0.86 | 0.39 | 1.17 | 0.24 |
| 1.2 | 2,11 | 1,56 | 2,54 | 2,25 | 1,57 | 0,30 | - 0.22 | 0.82 | - 0.81 | 0.42 | 1.16 | 0.25 |
| 1.3 | 2,11 | 1,53 | 2,47 | 2,24 | 1,57 | 0,33 | - 0.22 | 0.83 | - 0.76 | 0.44 | 1.14 | 0.25 |
| 1.4 | 2,11 | 1,51 | 2,45 | 2,23 | 1,55 | 0,34 | - 0.22 | 0.83 | - 0.71 | 0.48 | 1.12 | 0.26 |
| 1.5 | 2,10 | 1,50 | 2,44 | 2,23 | 1,53 | 0,33 | - 0.21 | 0.83 | - 0.66 | 0.51 | 1.11 | 0.27 |
| 1.6 | 2,09 | 1,49 | 2,42 | 2,22 | 1,53 | 0,33 | - 0.20 | 0.84 | - 0.61 | 0.54 | 1.10 | 0.27 |
| 1.7 | 2,08 | 1,49 | 2,40 | 2,20 | 1,52 | 0,32 | - 0.18 | 0.85 | - 0.57 | 0.57 | 1.10 | 0.27 |
| 1.8 | 2,06 | 1,50 | 2,37 | 2,15 | 1,51 | 0,32 | - 0.16 | 0.87 | - 0.53 | 0.60 | 1.11 | 0.27 |
| 1.9 | 2,05 | 1,52 | 2,36 | 2,10 | 1,50 | 0,31 | - 0.14 | 0.89 | - 0.50 | 0.61 | 1.12 | 0.26 |
| 2.0 | 2,05 | 1,54 | 2,36 | 2,05 | 1,50 | 0,32 | - 0.11 | 0.91 | - 0.48 | 0.63 | 1.15 | 0.25 |
| 2.1 | 2,04 | 1,56 | 2,38 | 2,00 | 1,49 | 0,33 | - 0.09 | 0.93 | - 0.47 | 0.64 | 1.18 | 0.24 |
| 2.2 | 2,04 | 1,59 | 2,39 | 1,96 | 1,49 | 0,33 | - 0.07 | 0.95 | - 0.47 | 0.64 | 1.21 | 0.23 |
| 2.3 | 2,03 | 1,60 | 2,37 | 1,92 | 1,48 | 0,34 | - 0.06 | 0.95 | - 0.48 | 0.63 | 1.24 | 0.22 |
| 2.4 | 2,03 | 1,61 | 2,35 | 1,89 | 1,47 | 0,35 | - 0.06 | 0.95 | - 0.49 | 0.62 | 1.27 | 0.21 |
| 2.5 | 2,03 | 1,62 | 2,33 | 1,86 | 1,46 | 0,35 | - 0.08 | 0.94 | - 0.52 | 0.60 | 1.29 | 0.20 |
| 2.6 | 2,02 | 1,61 | 2,32 | 1,85 | 1,45 | 0,35 | - 0.09 | 0.93 | - 0.55 | 0.58 | 1.31 | 0.19 |
| 2.7 | 2,02 | 1,61 | 2,31 | 1,83 | 1,44 | 0,36 | - 0.11 | 0.91 | - 0.58 | 0.56 | 1.33 | 0.18 |
| 2.8 | 2,01 | 1,60 | 2,31 | 1,82 | 1,43 | 0,36 | - 0.13 | 0.89 | - 0.61 | 0.54 | 1.34 | 0.18 |
| 2.9 | 2,00 | 1,59 | 2,32 | 1,83 | 1,41 | 0,36 | - 0.15 | 0.88 | - 0.64 | 0.52 | 1.35 | 0.18 |
| 3.0 | 2,00 | 1,58 | 2,33 | 1,84 | 1,39 | 0,37 | - 0.17 | 0.87 | - 0.67 | 0.51 | 1.36 | 0.18 |
| 3.1 | 2,01 | 1,57 | 2,35 | 1,86 | 1,38 | 0,37 | - 0.19 | 0.85 | - 0.69 | 0.49 | 1.36 | 0.17 |
| 3.2 | 2,02 | 1,55 | 2,37 | 1,84 | 1,36 | 0,38 | - 0.21 | 0.84 | - 0.72 | 0.47 | 1.36 | 0.17 |
| 3.3 | 2,02 | 1,54 | 2,38 | 1,81 | 1,34 | 0,38 | - 0.23 | 0.82 | - 0.74 | 0.45 | 1.36 | 0.17 |
| 3.4 | 2,03 | 1,52 | 2,39 | 1,78 | 1,32 | 0,38 | - 0.25 | 0.80 | - 0.78 | 0.44 | 1.36 | 0.17 |
| 3.5 | 2,04 | 1,50 | 2,38 | 1,75 | 1,31 | 0,38 | - 0.28 | 0.78 | - 0.81 | 0.42 | 1.36 | 0.17 |
| 3.6 | 2,05 | 1,48 | 2,36 | 1,72 | 1,29 | 0,38 | - 0.31 | 0.76 | - 0.84 | 0.40 | 1.36 | 0.17 |
| 3.7 | 2,06 | 1,46 | 2,30 | 1,71 | 1,28 | 0,38 | - 0.35 | 0.73 | - 0.87 | 0.38 | 1.36 | 0.17 |
| 3.8 | 2,06 | 1,43 | 2,24 | 1,69 | 1,27 | 0,38 | - 0.38 | 0.70 | - 0.90 | 0.37 | 1.35 | 0.18 |
| 3.9 | 2,06 | 1,41 | 2,22 | 1,69 | 1,26 | 0,38 | - 0.41 | 0.68 | - 0.92 | 0.36 | 1.34 | 0.18 |
| 4.0 | 2,05 | 1,39 | 2,21 | 1,68 | 1,25 | 0,37 | - 0.42 | 0.67 | - 0.92 | 0.36 | 1.33 | 0.18 |

**Table S1B: Mixed-model results for delta power during the light period following a torpor bout (Post-torpor at 25°C and 10°C) and the corresponding control period (Pre-torpor).**

Values are expressed as median (logarithm) ± interquartile range. Pre-torpor and Post-torpor at 25°C: n = 4, number of events = 13; Post-torpor at 10°C: n = 2, number of events = 8.

|  | Pre-torpor | | Post-torpor at 25°C | | Post-torpor at 10°C | | Pre-torpor vs  Post-torpor at 25°C | | Pre-torpor vs  Post-torpor at 10°C | | Post-torpor at 25°C vs Post-torpor at 10°C | |
| --- | --- | --- | --- | --- | --- | --- | --- | --- | --- | --- | --- | --- |
| Frequency  (Hz) | ***median*** | ***IQR*** | ***median*** | ***IQR*** | ***median*** | ***IQR*** | ***t*** | ***p-value*** | ***t*** | ***p-value*** | ***t*** | ***p-value*** |
| 0.5 | 2,98 | 1,32 | 3,44 | 0,45 | 1,74 | 0,56 | 2.82 | 0.005 | - 1.34 | 0.18 | 3.71 | < 0.001 |
| 0.6 | 2,94 | 1,23 | 3,35 | 0,45 | 1,70 | 0,47 | 2.76 | 0.006 | - 1.36 | 0.17 | 3.77 | < 0.001 |
| 0.7 | 2,86 | 1,14 | 3,27 | 0,48 | 1,71 | 0,44 | 2.68 | 0.007 | - 1.37 | 0.17 | 3.88 | < 0.001 |
| 0.8 | 2,80 | 1,05 | 3,23 | 0,50 | 1,71 | 0,40 | 2.60 | 0.01 | - 1.38 | 0.16 | 4.08 | < 0.0001 |
| 0.9 | 2,76 | 0,96 | 3,22 | 0,51 | 1,71 | 0,34 | 2.50 | 0.01 | - 1.40 | 0.16 | 4.27 | < 0.0001 |
| 1.0 | 2,74 | 0,89 | 3,20 | 0,52 | 1,70 | 0,29 | 2.43 | 0.02 | - 1.41 | 0.16 | 4.41 | < 0.0001 |
| 1.1 | 2,62 | 0,83 | 3,18 | 0,57 | 1,67 | 0,28 | 2.43 | 0.02 | - 1.41 | 0.16 | 4.45 | < 0.0001 |
| 1.2 | 2,54 | 0,80 | 3,13 | 0,60 | 1,65 | 0,28 | 2.48 | 0.01 | - 1.39 | 0.16 | 4.51 | < 0.0001 |
| 1.3 | 2,59 | 0,78 | 3,10 | 0,61 | 1,64 | 0,28 | 2.51 | 0.01 | - 1.36 | 0.17 | 4.55 | < 0.0001 |
| 1.4 | 2,61 | 0,77 | 3,08 | 0,62 | 1,60 | 0,28 | 2.54 | 0.01 | - 1.32 | 0.19 | 4.56 | < 0.0001 |
| 1.5 | 2,61 | 0,76 | 3,07 | 0,62 | 1,57 | 0,28 | 2.59 | 0.01 | - 1.27 | 0.20 | 4.53 | < 0.0001 |
| 1.6 | 2,58 | 0,75 | 3,05 | 0,62 | 1,56 | 0,28 | 2.65 | 0.008 | - 1.22 | 0.23 | 4.49 | < 0.0001 |
| 1.7 | 2,54 | 0,75 | 3,04 | 0,62 | 1,56 | 0,27 | 2.65 | 0.008 | - 1.16 | 0.24 | 4.44 | < 0.0001 |
| 1.8 | 2,47 | 0,83 | 3,02 | 0,62 | 1,55 | 0,22 | 2.73 | 0.006 | - 1.11 | 0.27 | 4.42 | < 0.0001 |
| 1.9 | 2,40 | 0,90 | 3,01 | 0,62 | 1,55 | 0,19 | 2.80 | 0.005 | - 1.06 | 0.29 | 4.40 | < 0.0001 |
| 2.0 | 2,33 | 0,98 | 3,00 | 0,64 | 1,55 | 0,16 | 2.87 | 0.004 | - 1.02 | 0.31 | 4.39 | < 0.0001 |
| 2.1 | 2,30 | 1,02 | 2,99 | 0,66 | 1,53 | 0,15 | 2.91 | 0.004 | - 0.98 | 0.33 | 4.39 | < 0.0001 |
| 2.2 | 2,27 | 1,03 | 2,98 | 0,68 | 1,52 | 0,13 | 2.94 | 0.003 | - 0.94 | 0.35 | 4.39 | < 0.0001 |
| 2.3 | 2,27 | 1,04 | 2,97 | 0,71 | 1,50 | 0,11 | 2.98 | 0.003 | - 0.92 | 0.36 | 4.42 | < 0.0001 |
| 2.4 | 2,26 | 1,06 | 2,97 | 0,72 | 1,47 | 0,10 | 3.03 | 0.002 | - 0.89 | 0.37 | 4.48 | < 0.0001 |
| 2.5 | 2,25 | 1,07 | 2,96 | 0,70 | 1,46 | 0,10 | 3.11 | 0.002 | - 0.86 | 0.39 | 4.56 | < 0.0001 |
| 2.6 | 2,24 | 1,07 | 2,96 | 0,67 | 1,46 | 0,10 | 3.19 | 0.001 | - 0.84 | 0.40 | 4.65 | < 0.0001 |
| 2.7 | 2,24 | 1,08 | 2,96 | 0,63 | 1,45 | 0,10 | 3.27 | 0.001 | - 0.81 | 0.42 | 4.73 | < 0.0001 |
| 2.8 | 2,25 | 1,09 | 2,96 | 0,59 | 1,46 | 0,08 | 3.32 | < 0.001 | - 0.79 | 0.43 | 4.77 | < 0.0001 |
| 2.9 | 2,28 | 1,27 | 2,96 | 0,56 | 1,46 | 0,07 | 3.34 | < 0.001 | - 0.77 | 0.44 | 4.78 | < 0.0001 |
| 3.0 | 2,32 | 1,33 | 2,96 | 0,55 | 1,46 | 0,06 | 3.33 | < 0.001 | - 0.76 | 0.45 | 4.75 | < 0.0001 |
| 3.1 | 2,37 | 1,31 | 2,97 | 0,55 | 1,46 | 0,08 | 3.30 | 0.001 | - 0.76 | 0.45 | 4.70 | < 0.0001 |
| 3.2 | 2,40 | 1,30 | 2,98 | 0,56 | 1,46 | 0,09 | 3.26 | 0.001 | - 0.77 | 0.44 | 4.64 | < 0.0001 |
| 3.3 | 2,42 | 1,27 | 2,99 | 0,58 | 1,45 | 0,10 | 3.21 | 0.001 | - 0.79 | 0.43 | 4.59 | < 0.0001 |
| 3.4 | 2,43 | 1,12 | 3,00 | 0,59 | 1,44 | 0,10 | 3.15 | 0.002 | - 0.82 | 0.41 | 4.54 | < 0.0001 |
| 3.5 | 2,42 | 1,06 | 3,00 | 0,61 | 1,43 | 0,10 | 3.09 | 0.002 | - 0.85 | 0.40 | 4.52 | < 0.0001 |
| 3.6 | 2,39 | 1,04 | 3,00 | 0,62 | 1,40 | 0,11 | 3.05 | 0.002 | - 0.87 | 0.38 | 4.52 | < 0.0001 |
| 3.7 | 2,35 | 1,01 | 2,96 | 0,63 | 1,38 | 0,11 | 3.02 | 0.003 | - 0.90 | 0.37 | 4.55 | < 0.0001 |
| 3.8 | 2,30 | 1,02 | 2,93 | 0,63 | 1,35 | 0,11 | 3.01 | 0.003 | - 0.93 | 0.35 | 4.59 | < 0.0001 |
| 3.9 | 2,23 | 1,01 | 2,90 | 0,64 | 1,32 | 0,14 | 3.02 | 0.003 | - 0.96 | 0.34 | 4.62 | < 0.0001 |
| 4.0 | 2,17 | 1,00 | 2,87 | 0,66 | 1,29 | 0,16 | 3.03 | 0.002 | - 0.98 | 0.33 | 4.63 | < 0.0001 |

|  | Pre-torpor | | Post-torpor at 25°C | | Post-torpor at 10°C | | Pre-torpor vs Post-torpor at 25°C | | Pre-torpor vs Post-torpor at 10°C | | Post-torpor at 25°C vs Post-torpor at 10°C | |
| --- | --- | --- | --- | --- | --- | --- | --- | --- | --- | --- | --- | --- |
| Frequency (Hz) | ***median*** | ***IQR*** | ***median*** | ***IQR*** | ***median*** | ***IQR*** | ***t*** | ***p-value*** | ***t*** | ***p-value*** | ***t*** | ***p-value*** |
| 0.5 | 3,46 | 0,68 | 3,95 | 0,91 | 2,48 | 1,49 | 1.25 | 0.21 | - 1.84 | 0.07 | 2.61 | 0.009 |
| 0.6 | 3,40 | 0,65 | 3,88 | 0,90 | 2,41 | 1,42 | 1.31 | 0.19 | - 1.90 | 0.06 | 2.72 | 0.007 |
| 0.7 | 3,33 | 0,63 | 3,83 | 0,90 | 2,32 | 1,35 | 1.39 | 0.17 | - 1.98 | 0.05 | 2.86 | 0.004 |
| 0.8 | 3,26 | 0,61 | 3,78 | 0,91 | 2,23 | 1,27 | 1.46 | 0.14 | - 2.08 | 0.04 | 3.05 | 0.002 |
| 0.9 | 3,19 | 0,59 | 3,73 | 0,93 | 2,13 | 1,19 | 1.53 | 0.13 | - 2.21 | 0.03 | 3.26 | 0.001 |
| 1.0 | 3,13 | 0,57 | 3,69 | 0,95 | 2,04 | 1,11 | 1.57 | 0.12 | - 2.34 | 0.02 | 3.48 | < 0.001 |
| 1.1 | 3,07 | 0,60 | 3,66 | 0,98 | 1,95 | 1,04 | 1.59 | 0.11 | - 2.49 | 0.01 | 3.71 | < 0.001 |
| 1.2 | 3,02 | 0,63 | 3,61 | 1,00 | 1,88 | 0,98 | 1.60 | 0.11 | - 2.62 | 0.009 | 3.91 | < 0.0001 |
| 1.3 | 2,98 | 0,61 | 3,55 | 1,02 | 1,82 | 0,93 | 1.59 | 0.11 | - 2.74 | 0.006 | 4.08 | < 0.0001 |
| 1.4 | 2,94 | 0,56 | 3,51 | 1,05 | 1,79 | 0,87 | 1.58 | 0.11 | - 2.84 | 0.004 | 4.36 | < 0.0001 |
| 1.5 | 2,92 | 0,58 | 3,47 | 1,06 | 1,76 | 0,85 | 1.56 | 0.12 | - 2.93 | 0.003 | 4.36 | < 0.0001 |
| 1.6 | 2,94 | 0,60 | 3,44 | 1,07 | 1,74 | 0,83 | 1.54 | 0.12 | - 3.00 | 0.003 | 4.47 | < 0.0001 |
| 1.7 | 2,91 | 0,59 | 3,43 | 1,08 | 1,72 | 0,82 | 1.53 | 0.13 | - 3.06 | 0.002 | 4.58 | < 0.0001 |
| 1.8 | 2,92 | 0,58 | 3,43 | 1,08 | 1,71 | 0,80 | 1.52 | 0.13 | - 3.09 | 0.002 | 4.69 | < 0.0001 |
| 1.9 | 2,93 | 0,56 | 3,42 | 1,06 | 1,70 | 0,77 | 1.51 | 0.13 | - 3.11 | 0.002 | 4.80 | < 0.0001 |
| 2.0 | 2,93 | 0,58 | 3,40 | 1,05 | 1,67 | 0,74 | 1.50 | 0.13 | - 3.12 | 0.002 | 4.92 | < 0.0001 |
| 2.1 | 2,92 | 0,62 | 3,38 | 1,03 | 1,65 | 0,69 | 1.50 | 0.13 | - 3.12 | 0.002 | 5.03 | < 0.0001 |
| 2.2 | 2,91 | 0,65 | 3,36 | 1,00 | 1,63 | 0,64 | 1.50 | 0.13 | - 3.12 | 0.002 | 5.14 | < 0.0001 |
| 2.3 | 2,90 | 0,67 | 3,34 | 0,98 | 1,61 | 0,60 | 1.51 | 0.13 | - 3.12 | 0.002 | 5.25 | < 0.0001 |
| 2.4 | 2,88 | 0,69 | 3,32 | 0,95 | 1,59 | 0,56 | 1.52 | 0.13 | -3.13 | 0.002 | 5.36 | < 0.0001 |
| 2.5 | 2,87 | 0,70 | 3,30 | 0,92 | 1,58 | 0,52 | 1.55 | 0.13 | - 3.13 | 0.002 | 5.46 | < 0.0001 |
| 2.6 | 2,85 | 0,71 | 3,29 | 0,89 | 1,56 | 0,48 | 1.58 | 0.12 | - 3.16 | 0.002 | 5.55 | < 0.0001 |
| 2.7 | 2,83 | 0,70 | 3,28 | 0,87 | 1,54 | 0,45 | 1.61 | 0.11 | - 3.17 | 0.002 | 5.63 | < 0.0001 |
| 2.8 | 2,81 | 0,70 | 3,27 | 0,84 | 1,53 | 0,43 | 1.64 | 0.11 | - 3.17 | 0.002 | 5.69 | < 0.0001 |
| 2.9 | 2,80 | 0,68 | 3,26 | 0,82 | 1,53 | 0,41 | 1.66 | 0.10 | - 3.17 | 0.002 | 5.73 | < 0.0001 |
| 3.0 | 2,79 | 0,65 | 3,25 | 0,80 | 1,52 | 0,39 | 1.67 | 0.10 | - 3.18 | 0.001 | 5.77 | < 0.0001 |
| 3.1 | 2,78 | 0,61 | 3,25 | 0,78 | 1,52 | 0,38 | 1.66 | 0.10 | - 3.19 | 0.001 | 5.80 | < 0.0001 |
| 3.2 | 2,77 | 0,57 | 3,25 | 0,77 | 1,52 | 0,38 | 1.66 | 0.10 | - 3.20 | 0.001 | 5.83 | < 0.0001 |
| 3.3 | 2,77 | 0,54 | 3,25 | 0,75 | 1,51 | 0,38 | 1.66 | 0.10 | - 3.22 | 0.001 | 5.85 | < 0.0001 |
| 3.4 | 2,76 | 0,52 | 3,26 | 0,74 | 1,51 | 0,39 | 1.65 | 0.10 | - 3.24 | 0.001 | 5.87 | < 0.0001 |
| 3.5 | 2,73 | 0,49 | 3,26 | 0,72 | 1,50 | 0,41 | 1.65 | 0.10 | - 3.26 | 0.001 | 5.88 | < 0.0001 |
| 3.6 | 2,71 | 0,47 | 3,27 | 0,71 | 1,48 | 0,42 | 1.66 | 0.10 | - 3.28 | 0.001 | 5.88 | < 0.0001 |
| 3.7 | 2,70 | 0,45 | 3,27 | 0,70 | 1,47 | 0,43 | 1.66 | 0.10 | - 3.29 | 0.001 | 5.87 | < 0.0001 |
| 3.8 | 2,70 | 0,44 | 3,28 | 0,70 | 1,46 | 0,43 | 1.67 | 0.10 | - 3.29 | 0.001 | 5.85 | < 0.0001 |
| 3.9 | 2,71 | 0,43 | 3,27 | 0,70 | 1,45 | 0,43 | 1.68 | 0.09 | - 3.29 | 0.001 | 5.82 | < 0.0001 |
| 4.0 | 2,71 | 0,43 | 3,27 | 0,70 | 1,43 | 0,42 | 1.68 | 0.09 | - 3.28 | 0.001 | 5.79 | < 0.0001 |

**Table S1C: Mixed-model results for delta power during the night period following a torpor bout (Post-torpor at 25°C and 10°C) and the corresponding control period (Pre-torpor).**

Values are expressed as median (logarithm) ± interquartile range. Pre-torpor: n = 4, number of events = 13; Post-torpor at 25°C: n = 4, number of events = 12; Post-torpor at 10°C: n = 2, number of events = 10.

**Table S1D: Mixed-model results for delta power during the light period the day after a torpor bout (Post-torpor at 25°C and 10°C) and the corresponding control period (Pre-torpor).**

Values are expressed as median (logarithm) ± interquartile range. Pre-torpor: n = 4, number of events = 13; Post-torpor at 25°C: n = 4, number of events = 12; Post-torpor at 10°C: n = 2, number of events = 8.

|  | Pre-torpor | | Post-torpor at 25°C | | Post-torpor at 10°C | | Pre-torpor vs Post-torpor at 25°C | | Pre-torpor vs Post-torpor at 10°C | | Post-torpor at 25°C vs Post-torpor at 10°C | |
| --- | --- | --- | --- | --- | --- | --- | --- | --- | --- | --- | --- | --- |
| Frequency  (Hz) | ***median*** | ***IQR*** | ***median*** | ***IQR*** | ***median*** | ***IQR*** | ***t*** | ***p-value*** | ***t*** | ***p-value*** | ***t*** | ***p-value*** |
| 0.5 | 2,60 | 1,22 | 3,32 | 1,07 | 1,47 | 0,31 | 1.26 | 0.21 | - 1.18 | 0.24 | 2.81 | 0.005 |
| 0.6 | 2,56 | 1,17 | 3,27 | 1,11 | 1,50 | 0,27 | 1.30 | 0.20 | - 1.16 | 0.25 | 2.84 | 0.005 |
| 0.7 | 2,50 | 1,12 | 3,21 | 1,17 | 1,53 | 0,25 | 1.32 | 0.19 | - 1.15 | 0.25 | 2.87 | 0.004 |
| 0.8 | 2,44 | 1,07 | 3,15 | 1,24 | 1,56 | 0,24 | 1.34 | 0.18 | - 1.17 | 0.25 | 2.91 | 0.004 |
| 0.9 | 2,38 | 1,02 | 3,08 | 1,30 | 1,57 | 0,24 | 1.35 | 0.18 | - 1.20 | 0.24 | 2.95 | 0.003 |
| 1.0 | 2,31 | 0,98 | 3,01 | 1,35 | 1,57 | 0,26 | 1.34 | 0.18 | - 1.23 | 0.23 | 3.00 | 0.003 |
| 1.1 | 2,24 | 0,93 | 2,95 | 1,39 | 1,56 | 0,27 | 1.34 | 0.18 | - 1.27 | 0.22 | 3.06 | 0.002 |
| 1.2 | 2,18 | 0,89 | 2,92 | 1,40 | 1,55 | 0,28 | 1.34 | 0.18 | - 1.31 | 0.20 | 3.10 | 0.002 |
| 1.3 | 2,12 | 0,88 | 2,91 | 1,40 | 1,55 | 0,28 | 1.34 | 0.18 | - 1.34 | 0.19 | 3.14 | 0.002 |
| 1.4 | 2,07 | 0,87 | 2,90 | 1,39 | 1,54 | 0,28 | 1.34 | 0.18 | - 1.37 | 0.18 | 3.16 | 0.002 |
| 1.5 | 2,04 | 0,86 | 2,88 | 1,37 | 1,52 | 0,29 | 1.35 | 0.18 | - 1.39 | 0.17 | 3.05 | 0.002 |
| 1.6 | 2,02 | 0,86 | 2,87 | 1,35 | 1,50 | 0,30 | 1.35 | 0.18 | - 1.39 | 0.17 | 2.70 | 0.007 |
| 1.7 | 2,00 | 0,84 | 2,85 | 1,34 | 1,48 | 0,31 | 1.35 | 0.18 | - 1.39 | 0.16 | 2.57 | 0.01 |
| 1.8 | 2,01 | 0,82 | 2,84 | 1,33 | 1,47 | 0,33 | 1.34 | 0.19 | - 1.39 | 0.16 | 2.29 | 0.02 |
| 1.9 | 2,04 | 0,83 | 2,81 | 1,32 | 1,46 | 0,37 | 1.32 | 0.19 | - 1.39 | 0.16 | 2.16 | 0.03 |
| 2.0 | 2,07 | 0,84 | 2,79 | 1,31 | 1,46 | 0,41 | 1.30 | 0.20 | - 1.39 | 0.16 | 2.07 | 0.04 |
| 2.1 | 2,09 | 0,84 | 2,76 | 1,32 | 1,46 | 0,44 | 1.27 | 0.21 | - 1.40 | 0.16 | 1.99 | 0.05 |
| 2.2 | 2,10 | 0,84 | 2,74 | 1,35 | 1,46 | 0,48 | 1.23 | 0.23 | - 1.42 | 0.16 | 1.93 | 0.05 |
| 2.3 | 2,09 | 0,83 | 2,72 | 1,39 | 1,45 | 0,52 | 1.20 | 0.24 | - 1.44 | 0.15 | 1.87 | 0.06 |
| 2.4 | 2,07 | 0,83 | 2,72 | 1,42 | 1,45 | 0,56 | 1.17 | 0.25 | - 1.48 | 0.14 | 1.83 | 0.07 |
| 2.5 | 2,03 | 0,83 | 2,72 | 1,46 | 1,45 | 0,60 | 1.16 | 0.25 | - 1.51 | 0.13 | 1.79 | 0.07 |
| 2.6 | 2,01 | 0,83 | 2,72 | 1,48 | 1,44 | 0,64 | 1.16 | 0.24 | - 1.54 | 0.12 | 1.77 | 0.08 |
| 2.7 | 2,00 | 0,83 | 2,73 | 1,49 | 1,43 | 0,68 | 1.17 | 0.24 | - 1.57 | 0.12 | 1.75 | 0.08 |
| 2.8 | 2,00 | 0,82 | 2,73 | 1,50 | 1,42 | 0,71 | 1.18 | 0.23 | - 1.59 | 0.11 | 1.75 | 0.08 |
| 2.9 | 1,99 | 0,81 | 2,72 | 1,52 | 1,41 | 0,75 | 1.20 | 0.23 | - 1.60 | 0.10 | 1.75 | 0.08 |
| 3.0 | 1,98 | 0,81 | 2,71 | 1,53 | 1,39 | 0,78 | 1.21 | 0.23 | - 1.62 | 0.10 | 1.76 | 0.08 |
| 3.1 | 1,97 | 0,80 | 2,70 | 1,54 | 1,38 | 0,81 | 1.22 | 0.22 | - 1.63 | 0.10 | 1.76 | 0.08 |
| 3.2 | 1,96 | 0,79 | 2,69 | 1,55 | 1,36 | 0,84 | 1.22 | 0.22 | - 1.64 | 0.10 | 1.76 | 0.08 |
| 3.3 | 1,98 | 0,78 | 2,68 | 1,56 | 1,35 | 0,87 | 1.23 | 0.22 | - 1.66 | 0.10 | 1.76 | 0.08 |
| 3.4 | 2,01 | 0,77 | 2,68 | 1,57 | 1,34 | 0,90 | 1.24 | 0.21 | - 1.67 | 0.10 | 1.75 | 0.08 |
| 3.5 | 2,03 | 0,77 | 2,68 | 1,58 | 1,32 | 0,93 | 1.26 | 0.21 | - 1.69 | 0.09 | 1.75 | 0.08 |
| 3.6 | 2,03 | 0,76 | 2,68 | 1,60 | 1,31 | 0,95 | 1.27 | 0.20 | - 1.71 | 0.09 | 1.74 | 0.08 |
| 3.7 | 2,01 | 0,76 | 2,67 | 1,61 | 1,30 | 0,97 | 1.28 | 0.20 | - 1.74 | 0.08 | 1.73 | 0.08 |
| 3.8 | 1,99 | 0,77 | 2,66 | 1,63 | 1,29 | 0,98 | 1.29 | 0.20 | - 1.76 | 0.08 | 1.73 | 0.08 |
| 3.9 | 1,95 | 0,76 | 2,66 | 1,66 | 1,28 | 0,99 | 1.31 | 0.19 | - 1.77 | 0.08 | 1.73 | 0.08 |
| 4.0 | 1,91 | 0,77 | 2,65 | 1,69 | 1,27 | 1,00 | 1.32 | 0.19 | - 1.79 | 0.07 | 1.73 | 0.08 |
